# SIGNIFICANCE OF SECOND YEAR MEDICAL STUDENTS PARTICIPATING IN THE 48-HOUR HOSPICE HOME IMMERSION PROJECT, 2017–2018

**DOI:** 10.1093/geroni/igac059.2024

**Published:** 2022-12-20

**Authors:** Samuel Hanlon, Benjamin Packard, Marilyn Gugliucci

**Affiliations:** University of New England College of Osteopathic Medicine, Biddeford, Maine, United States; University of New England College of Osteopathic Medicine, Biddeford, Maine, United States; University of New England College of Osteopathic Medicine, Biddeford, Maine, United States

## Abstract

Medical education on palliative medicine and end-of-life care is generally lacking in the medical curricula. The University of New England College of Osteopathic Medicine (UNECOM) Learning by Living: 48 Hour Hospice Home Immersion Project is an immersion-based learning model whereby UNECOM 2nd year students live in an18-bed acute care hospice house to care for dying patients, provide family support, and conduct post-mortem care. This project determined if and in what ways immersion experiences were valuable in augmenting student medical end-of-life care education during AY 2017-2018.Retrospective ethnographic/autobiographic data were analyzed from the eight randomly selected student hospice immersion journals (approx. 200 pages) who participated during academic year 2017-18. Pre-fieldwork, fieldwork, post-fieldwork journals were reviewed and analyzed using manual content analysis followed by NVivo 12+ analysis. Thematic coding resulted in representative quotes, key words, and native concepts. Inter-rater reliability was established with the use of a codebook and agreed upon thematic definitions. Four key themes included: Subversion of End of life (EOL) Expectations; Character Development/Introspection; Exposure to Diverse Cultural/Spiritual Perspectives; and Skills to Bring into Future Practice. Proximity to death/dying resulted in reflections on values and priorities, and a renewed sense for compassionate patient care. Students developed skills for future practice, including competency in EOL and post-mortem care, navigating difficult, emotionally laden family dynamics, and contributing to an interprofessional staff team even in uncomfortable situations. This immersion positively affected student perspectives about death and end-of-life care; creating life-altering experiences in patient-centered-care. Students stated significant impacts to employ as a physician.

